# Numerical Analysis of a Dental Zirconium Restoration and the Stresses That Occur in Dental Tissues

**DOI:** 10.1155/2019/1049306

**Published:** 2019-09-05

**Authors:** Rosa Alicia Hernández-Vázquez, Guillermo Urriolagoitia-Sosa, Rodrigo Arturo Marquet-Rivera, Beatriz Romero-Ángeles, Octavio-Alejandro Mastache-Miranda, G. Guillermo Urriolagoitia-Calderón

**Affiliations:** Instituto Politécnico Nacional, Escuela Superior de Ingeniería Mecánica y Eléctrica, Sección de Estudios de Posgrado e Investigación, Unidad Profesional Adolfo López Mateos “Zacatenco, ” Avenida Instituto Politécnico Nacional, S/n Edificio 5, 2do. Piso, Col. Lindavista, C.P. 07320 Ciudad de México, Mexico

## Abstract

When it is about restorative dental materials, aesthetics is traditionally preferred. This has led to the selection of materials very visually similar to the enamel, but unfortunately, their mechanical properties are not similar. This often translates into disadvantages than advantages. In the present work, a comparison is made of the stresses that occur during dental occlusion (dental bit) in a healthy dental organ and those that are generated in a dental organ with a dental zirconium restoration. Numerical simulation was carried out by means of the Finite Element Method, in computational biomodels, from Cone-Beam Tomography, to obtain the stresses generated during dental occlusion. It was found that the normal and *von Mises* stresses generated are substantially greater in the molar with restoration compared to those produced in the healthy molar. In addition, the normal function of the enamel and dentin to disperse these stresses to prevent them from reaching the pulp is altered. Therefore, it is necessary to analyze the indiscriminate use of this restoration material and consider other aspects, in addition to aesthetics and biocompatibility for the choice of restorative materials such as *biomechanical compatibility*.

## 1. Introduction

Today's dentistry is focused on achieving its transformation, from being an area of therapeutic medicine to becoming a preventive health area. However, it is still on track to achieve that goal. The odontological task is mainly of restorative type. This restorative nature of dentistry is due to the high incidence of caries and its consequences. It has been reported that 95% of the world population suffers from or has suffered tooth caries. The rate of caries recidivism or the restorative treatment by itself is not a resolutive therapy [1]. Facing these aspects, the dentist must select the treatment and restorative material suitable for each patient. They should consider the behavior of the tooth to rehabilitate and restore it considering different variables, such as masticatory forces and the type of pathology that occurs. In the case of carious lesions, it is necessary to consider the degree and location of the defect, the evolution time, the degree of aggressiveness, the amount of affected tissue, and the healthy tissue remaining.

A fundamental aspect that should be considered is that dental tissue affected by caries has undergone variations in its biological, physical, chemical, and mechanical properties, due to the caries process and mastication. There is evidence that, in dental tissues affected by caries, there is a *stiffening* phenomenon of both the affected tissue and the healthy remnant, which makes the tissues more fragile by mechanical means [2, 3]. This agrees with general dentistry knowledge that a dental organ with caries is more prone to fracture during chewing, than a healthy one. And fundamental aspects should be considered for the selection of the type of restoration and restorative material to be applied. A little less than 20 years ago, the use of various ceramic materials for the manufacture of dental restorations was implemented. These materials have properties with greater similarities to those of dental tissues in terms of strength, aesthetics, and biocompatibility [4].

For the dental community, the hardness and resistance of a restorative material are of utmost importance, since there exists a paradigm that this mechanical property allows the restoration to work efficiently and for a long period. This conceptualization is not completely adequate. Ceramic materials have a mechanical behavior with less predictability than metals [5]. In addition, ceramics are hard but fragile [6]. There are concepts that can be contradictory for some sectors of the general dental community. Nowadays, restorations made with zirconium (commonly called zirconia, dental zirconium, dental zirconia, dental zirconia, or dental zirconium) are currently in great use [7]. This material has generated a considerable interest for its application in dentistry, due to properties that are considered ideal. In various articles of dental journals, it is said that it is a highly aesthetic material with an acceptable lifespan (between three and five years) and with an average success rate of 94% [8–11].

In terms of physical-mechanical properties, zirconium has great standout advantages such as high values of toughness, great hardness, wear resistance, good frictional behavior, good electrical insulation, low thermal conductivity, and resistance to corrosion (substances acids and alkaline), which makes it an ideal material [9]. It is also mentioned that it has a modulus of elasticity like steel and a coefficient of thermal expansion like iron [12]. Values are higher than those of the tooth enamel and dentin, so the hardness and rigidity are greater than those of both dental tissues. Although dental zirconium and dental tissues are materials of the same nature (hard and fragile) to have ranges so distant in the values of their mechanical properties (elasticity module mainly), their mechanical behavior also varies. Their stress-strain graphs, although they behave similarly (rigid/hard materials), cannot be identified as similar. The stress necessary to cause a deformation in the dental zirconium is greater than those required for the enamel and even greater for the dentin [13].

Several studies have established that the use of this material causes the chipping of the coating ceramic, central fractures of the restored dental organ, and the abrasion and wear of the antagonist teeth [14]. On the other hand, it is also mentioned that, for its placement, it requires greater wear of the healthy remaining tissues of the organ to be restored and, during the chewing process, the action of moisture in the oral cavity microfractures can occur. It is common to find that patients with this kind of restorations tend to return for consultation; this is because their restored tooth has been fractured or pain is present when chewing. In some cases, the opposing tooth to the restoration is the one that presents pain during the mastication or worst when fracture occurs [15–22]. Another situation to consider is the one in which the extent of this material can alter the normal function of dental tissues. In a healthy tooth, the masticatory forces act on the enamel, the material of the tooth which is a hard but fragile tissue. These loads pass through the enamel and are received by the dentin, which is a specialized connective tissue with a greater amount of collagen than the enamel, so it is more elastic. This tissue supports the enamel and compensates for its fragility preventing it from easily fracturing. In addition, the dentin is responsible for sending the loads and stress that are produced by the masticatory forces towards the periodontal ligament and the alveolar bone; this function is fundamental for the protection of the pulp. In this manner, the dental pulp does not receive any type of mechanical agent (load or stress) that could cause irritation or inflammation in it.

As already mentioned, it is imperative to consider the nature and mechanical behavior of both dental tissues and restorative material, in this case, dental zirconium. The main factor to consider is the rigidity of the material, understanding rigidity as the resistance of a material to undergo deformations; hence, it is granted in turn the property of being hard but fragile. By not having the ability to deform, the material fails, and the fracture ensues. The dentin is capable of solving the enamel's rigidity and supports its inability to deform, preventing the enamel from failing or fracturing. In this same way, the occlusal loads and stresses that the enamel backs receive are dissipated by the dentin to avoid reaching the pulp tissue.

When a restoration with dental zirconium is placed, this material exceeds the hardness and rigidity of the dental tissues, in a dental organ with a history of caries; these remaining tissues have undergone an alteration in their properties, but not only chemical and biological but also mechanical, so the repercussions are greater. Dental zirconium is a very hard and rigid material that is placed in the enamel and dentin; a tissue that has been *stiffened* and that has lost its supporting tissue, the dentin causes the loads and stresses to increase and reach areas in which they should be present. Therefore, the dentin would be exceeded in its function of solving the rigidity and the incapacity of the deformation. In this way, although the dental zirconium would not suffer faults, the remaining enamel would do so due to the differences between the mechanical behaviors of both materials. In addition, the dentin would not be able to protect the dental pulp, causing it to receive loads and stresses that should not be present.

The present work shows the reactions that occur in the pulp tissue when a dental zirconium restoration is used, due to the differences in mechanical behavior described above, which modify the symbiotic or synergic relationship between the dental tissues. This is done through linear-elastic numerical analysis by means of the application of the Finite Element Method, from which high-biofidelity biomodels were used [23, 24].

## 2. Materials and Methods

**Figure 1:**
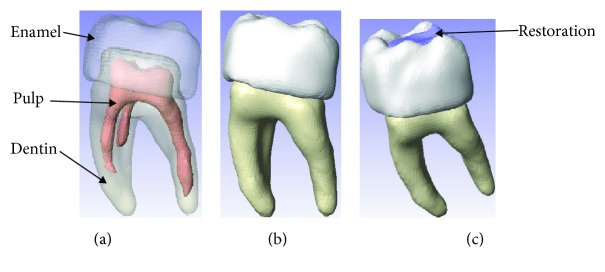
The molar: (a) three tissues, (b) healthy molar, and (b) molar dental zirconium restoration.

To find reactions and stress fields that arise in dental tissues through numerical analysis, two study cases were considered: Case 1—a control case, with a healthy lower first molar, and Case 2—a lower first molar with a history of second-degree caries on the occlusal face. It was restored with an *inlay* of dental zirconium. The biomodels corresponding to each case were generated from 3D imaging, by means of a Digital Volumetric Tomography (DVT) of the maxilla and mandible with the Computed Tomography System Cone Beam (CTCB), to obtain *DICOM* files. With these files and using a methodology developed by the authors in previous works [2, 25], these biomodels have high morphological and morphometric biofidelity; three tissues are considered for the molar: enamel, dentin, and pulp and the dental restoration (Figure 1).

For the numerical analyses, the tissues and dental zirconium of the biomodels are considered materials that present a linear, elastic, and continuous behavior, and their internal structure is considered to be isotropic and homogeneous. The boundary conditions are established at the dental rear zone of the dental roots; the displacements and rotations in the directions of the *X*, *Y*, and *Z* axes are restricted in this region. The properties of the materials for the simulation are presented in Table 1 [26–30].

A load was applied in the form of pressure on the occlusal area of the biomodels, to simulate the dental occlusion. The magnitude of the applied load is 150 N/mm^2^ which corresponds to the biting force that is established between both molars, which is distributed locally on the application area in the form of a pressure. It is important to mention that the bite contact (dental occlusion) is being simulated and analyzed, not the chewing process; that is why, only a single load that corresponds to this phenomenon is applied [31–34]. The contacts between the tissues and the restoration were considered for the analysis performed. The detailed methodology to obtain the biomodel, from the three-dimensional images of the tomography, to the boundary conditions and mesh refinement, among others, is the same with those used in the research and publications developed previously by the authors [2, 3, 23, 35] (Table 2).

The strain, displacements, normal stresses, shear stress, and *von Mises* stresses were analyzed during the application of the pressure that simulates dental bite or occlusion. However, for the purposes of this work, only the results obtained for nominal and *von Mises* stresses are shown. It should be mentioned that *von Mises* stresses are not considered here a failure criterion (which is mainly applicable to ductile materials) but a unique nondirectional value that allows to have a global criterion on the load at each tooth point, since it is obtained from the deformation energy. Several authors use it as a criterion to evaluate restorations [30, 36, 37].

## 3. Results

The results obtained for each case are shown in Tables 3–5 and Figures 2–13.

Figure 3 shows the normal stresses on the *X* axis, Figure 4 shows the normal stresses on the *Y* axis, Figure 5 shows the normal stresses on the *Z* axis, and Figure 6 shows the *von Mises* stresses in enamel for both cases.

Figure 6 shows the normal stresses on the *X* axis, Figure 7 shows the normal stresses on the *Y* axis, Figure 8 shows the normal stresses on the *Z* axis, and Figure 9 shows the *von Mises* stresses in the dentin for both cases.

Figure 10 shows the normal stresses on the *X* axis, Figure 11 shows the normal stresses on the *Y* axis, Figure 12 shows the normal stresses on the *Z* axis, and Figure 13 shows the *von Mises* stresses in the pulp for both cases.

Tables 3–5 show the results obtained from the numerical simulations carried out.

Table 3 shows that the nominal stresses generated by dental occlusion on the enamel are greater in the restored molar than the healthy molar. In the *X* axis, in the healthy molar, the maximum stresses are 0.0025 Pa in tension and in the restored molar they are -17.41 × 10^6^ Pa (-17.41 MPa/-17,410,000.00 Pa) in compression. In the *Y* axis, in the healthy molar, the maximum stresses are -0.0025 Pa and in the restored molar they are -26.63 × 10^6^ Pa (-26.63 MPa/-26,630,000.00 Pa) in compression for both cases. In the *Z* axis, in the healthy molar, the maximum stresses are 0.0096 Pa in tension and in the restored molar they are -47.90 × 10^6^ Pa (-26.63 MPa/-26,630,000.00 Pa) in compression. On the other hand, the stresses of *von Mises* are greater in the restored molar (40.28 × 10^6^ Pa) (40.28 MPa/40,280,000 Pa) in relation to the healthy molar that presents 0.0124 Pa.

Table 4 shows the same phenomenon described above, the nominal forces generated by dental occlusion on the dentin are greater in the restored molar than in the healthy molar but change the type of stress (tension or compression). In the *X* axis, in the healthy molar, the maximum stresses are -0.0015 Pa and in the restored molar they are -8.16 × 10^6^ Pa (-8.16 MPa/-8,160,000.00 Pa) both in compression. In the *Y* axis, in the healthy molar, the maximum stresses are 0.0015 Pa in tension and in the restored molar they are -6.33 × 10^6^ Pa (-6.33 MPa/-6,330,000.00 Pa) in compression. In the *Z* axis, in the healthy molar, the maximum stresses are -0.0026 Pa and in the restored molar are -28.90 × 10^6^ Pa (-28.90 MPa/-28.900,000.00 Pa) in compression for both cases. The same happens for the *von Mises* stresses, they are greater in the molar restored (27.65 × 10^6^ Pa) (28.90 MPa/28,900,000 Pa) in relation to the healthy molar that presents 0.0027 Pa.

Table 5 shows that the main hypothesis of the work is correct: While in the healthy molar, the stresses that are presented are negligible (almost zero), and in the restored molar, there are stresses of considerable value. As shown in Figure 13, the healthy molar presents the maximum stresses of -0.0006 Pa on the *X* axis, -0.0007 Pa on the *Y* axis, and -0.0004 for the *Z* axis; all of them in compression are consistent with the acting agent (occlusal load) in practically all of the pulp tissues. This is because the loads and stresses were dissipated by the dentin. On the other hand, it is important to mention that the critical areas where the maximum stresses are presented are due to the geometry of the tissue that, due to its anatomy, generate stress concentrators. The *von Mises* stresses present a maximum of 0.0007 Pa.

In contrast, the restored molar presents the maximum stresses of 4.69 × 10^6^ Pa (4.69 MPa/4,690,000 Pa) on the *X* axis, 4.79 × 10^6^ Pa (4.97 MPa/4,970,000 Pa) on the *Y* axis, and 11.01 × 10^6^ Pa (11.01 MPa/11,010,000) on the *Z* axis, in tension for the 3 axes. As for the *von Mises* stresses, the maximum values are 5.29 × 10^6^ Pa (5.29 MPa/5,290,000 Pa). Therefore, stresses are being present in a tissue where they should not be.

## 4. Discussion

Dental tissues are highly specialized; dental enamel is a tissue that must withstand masticatory forces, making it a very hard material. Within the human body, it certainly is the hardest material produced by the organism, a hard material presenting a high resistance to be penetrated or scratched making the material to have little or none ductility at all with almost no tolerance to deformation which makes it a fragile material. A hard material is capable to withstand high loads with virtually zero deformation. These means that if a high load is applied to a hard material, it will have a high resistance to support this load when a deformation is required; since this material does not have this property, then it suddenly fails, and a fracture is produced.

In order to mitigate this situation, the tooth has a support tissue that is not as hard as enamel, being more ductile and elastic; this tissue is the dentin. This tissue contains a greater amount of collagen, which gives it greater ductility and elasticity. The dentin, although it is a hard tissue-like bone, is able to withstand as much load as the enamel does, even if it is not the one that directly receives the total masticatory loads; by working in synergy with enamel, it reduces its fragility and helps to distribute the loads. As already mentioned, the enamel receives the loads and masticatory forces that are distributed throughout the occlusal surface, which contacts the food at the time of chewing. The dentin functions as a buffer tissue to deal with these mechanical agents, mitigating the fragility of the enamel and redirecting the forces and masticatory loads towards the periodontal ligament and the alveolar bone. In this way, the dentin fulfills the function of providing support to the enamel and at the same time protecting the enamel and mainly the pulp.

At this point, it would be important to ask how appropriate it is to consider a material harder than enamel to replace it and make dental restorations. Considering also that generally, when a restoration is required, it is because both the enamel and dentin have been lost, which is a fundamental tissue for the proper functioning of the enamel itself. With this loss, a greater *stiffness* of the remaining enamel has been found in previous studies [3], so when restoring with dental zirconium, this is not working symbiotically or synergistically with the dental tissues, as they do so naturally between them. This is why the tensions generated in the dental organ with the restoration are greater. The dentin cannot meet the demand of the material to cover its inability to deform which can cause faults in the remaining enamel and in the transmission of stresses to the pulp tissue.

**Figure 2:**
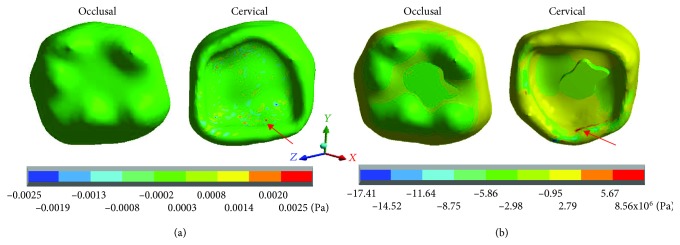
Enamel normal stresses on the *X* axis: (a) healthy molar and (b) molar with dental zirconium restoration.

**Figure 3:**
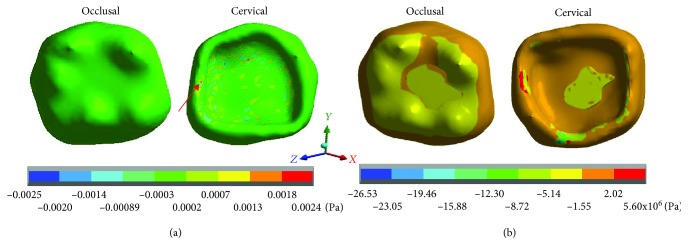
Enamel normal stresses on the *Y* axis: (a) healthy molar and (b) molar with dental zirconium restoration.

**Figure 4:**
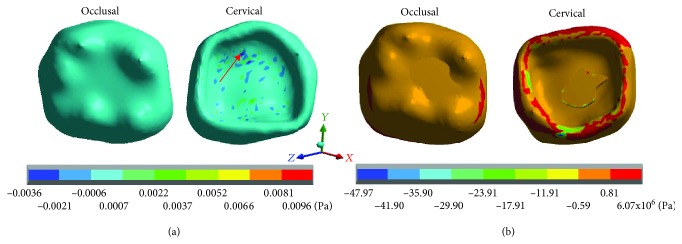
Enamel normal stresses on the *Z* axis: (a) healthy molar and (b) molar with dental zirconium restoration.

**Figure 5:**
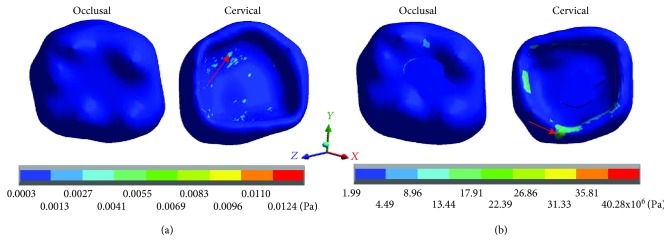
Enamel *von Mises* stresses: (a) healthy molar and (b) molar with dental zirconium restoration.

**Figure 6:**
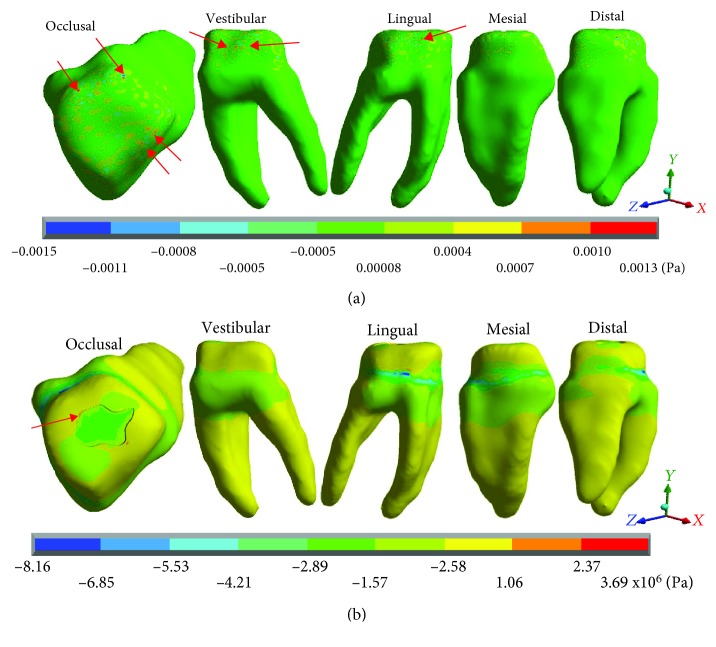
Dentin normal stresses on the *X* axis: (a) healthy molar and (b) molar with dental zirconium restoration.

**Figure 7:**
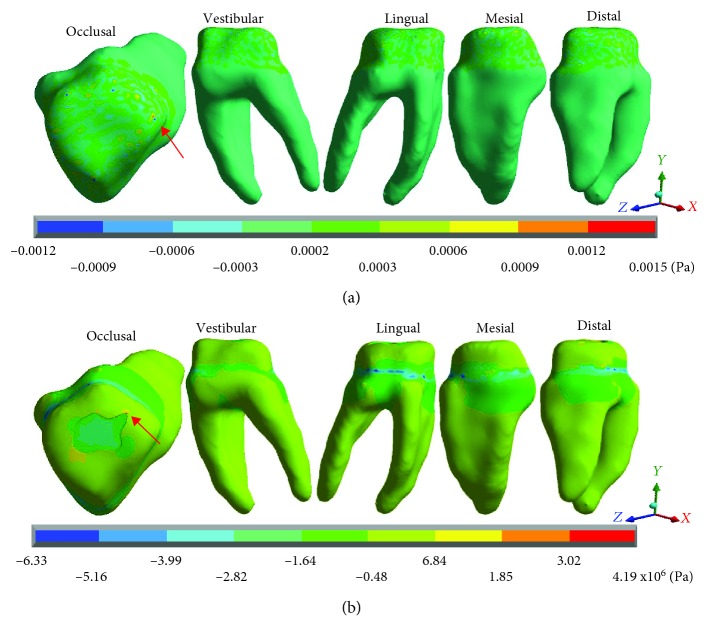
Dentin normal stresses on the *Y* axis: (a) healthy molar and (b) molar with dental zirconium restoration.

**Figure 8:**
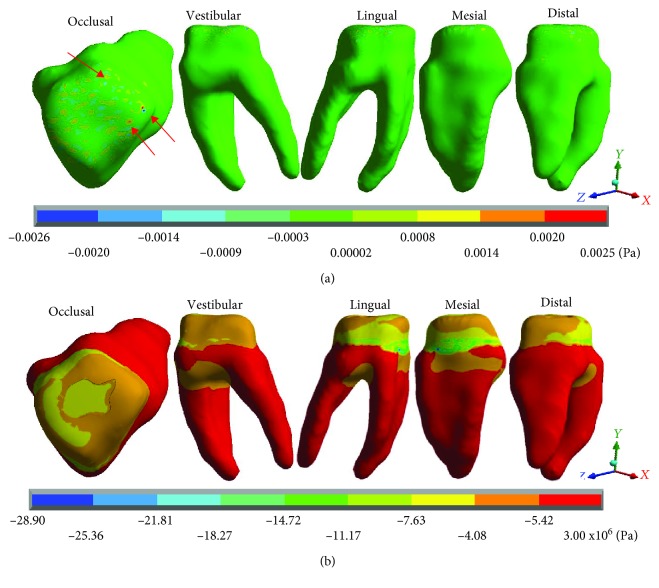
Dentin normal stresses on the *Z* axis: (a) healthy molar and (b) molar with dental zirconium restoration.

**Figure 9:**
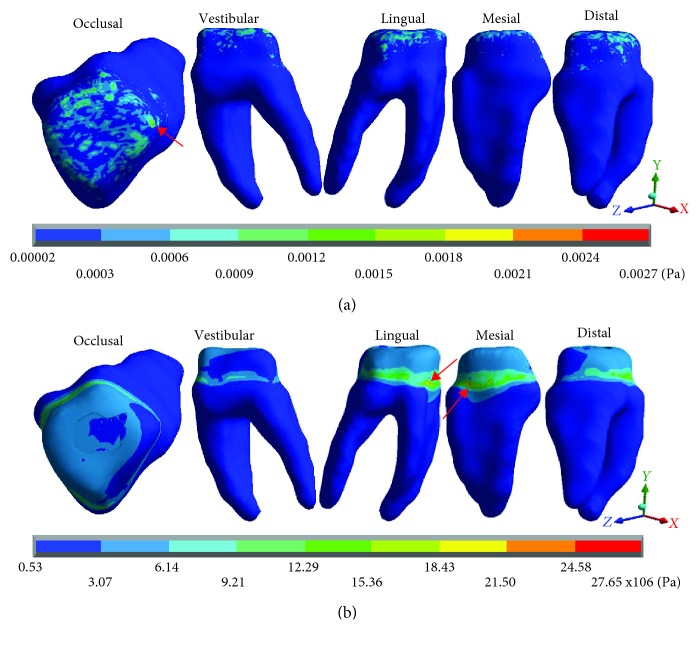
Dentin *von Mises* stresses: (a) healthy molar and (b) molar with dental zirconium restoration.

**Figure 10:**
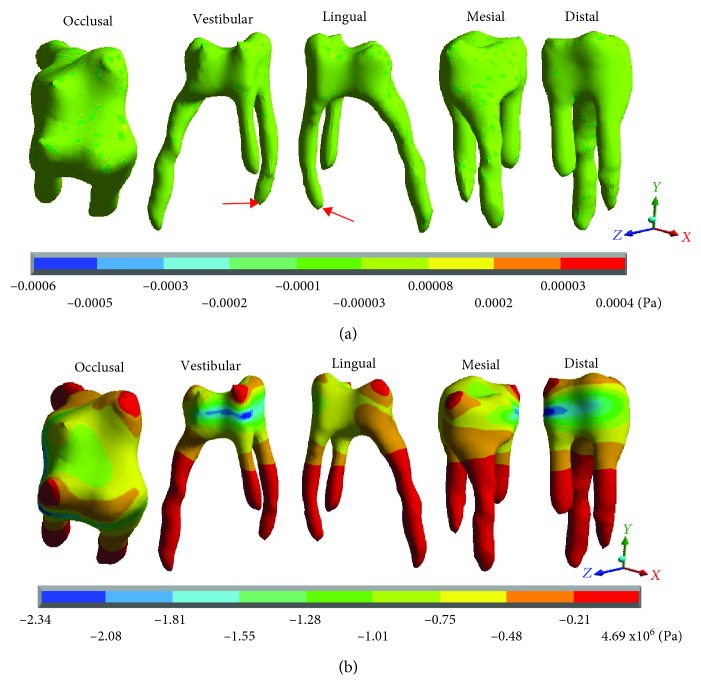
Pulp normal stresses on the *X* axis: (a) healthy molar and (b) molar with dental zirconium restoration.

**Figure 11:**
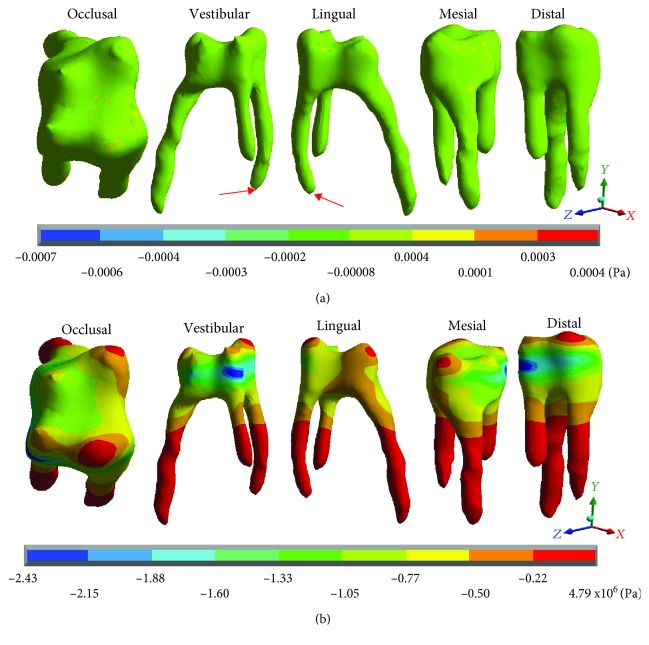
Pulp normal stresses on the *Y* axis: (a) healthy molar and (b) molar with dental zirconium restoration.

**Figure 12:**
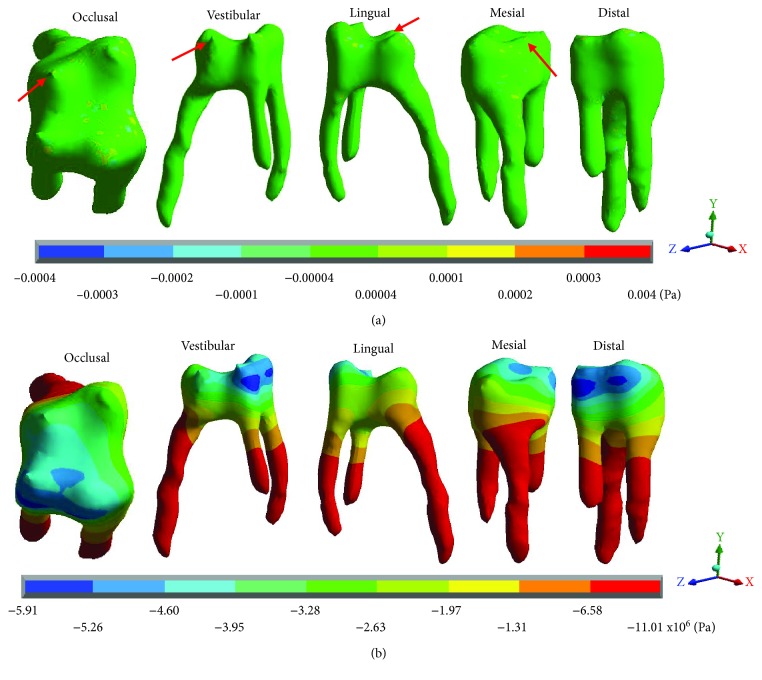
Pulp normal stresses on the *Z* axis: (a) healthy molar and (b) molar with dental zirconium restoration.

**Figure 13:**
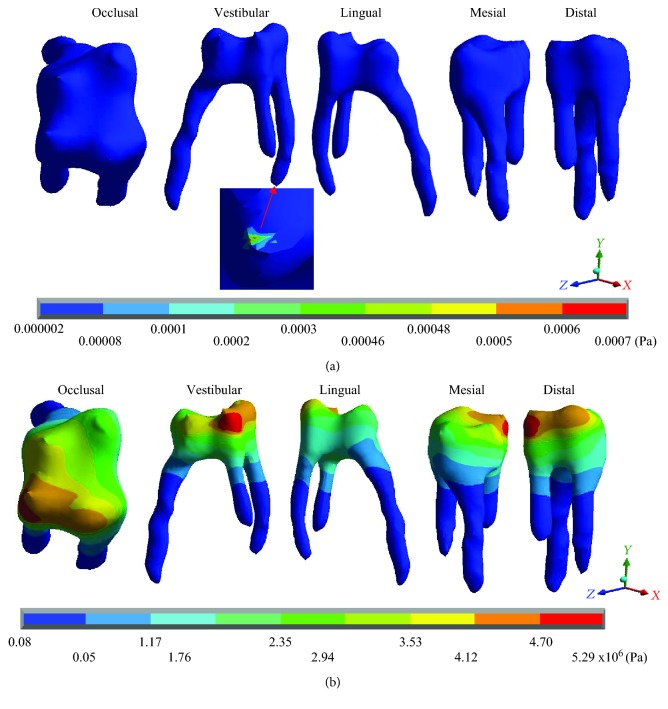
Pulp *von Mises* stresses: (a) healthy molar and (b) molar with dental zirconium restoration.

In the analyses carried out in the present work, in Table 2, it is possible to observe that the normal stresses in the three axes and those of *von Mises*, generated in the molar that has the restoration, are significantly increased in comparison to those of the healthy molar in the zones where the reactions are presented. The major critical zones are located mainly in the zone of the amelodentinous junction and in the pulp (Figures 2–9). In a general way, in the Control Case, the normal stresses on the pulp are practically null (Figure 3). In Case 2, they are much larger and more significant stresses (Figure 9), which indicate that the role of the dentin in preventing stresses reaching the pulp was nullified, because it has overcome its ability to support the rigidity or resistance to deformation, which presents the restoration. This could be related to the structural integrity of the molar for each case, as per the modified geometry in the restoration, but due to these results, this could be interpreted that this is because the mechanical properties of the tissues that are being replaced are not sufficiently similar.

Also, with these results, it is possible to prove numerically that the dental zirconium is too rigid, to be considered one and by itself, as a totally adequate material to replace lost dental tissues. These statements were also based on the clinical observation of the patients, where it is reported that the teeth that are antagonistic to the restorations are worn out by chewing, that the healthy remaining tissues that surround the restoration are fractured, and that there is pain when chewing.

## 5. Conclusions

Since its origins in dentistry, metal restorations had been the first option. However, the types of materials could not be fully biocompatible and could compromise the integrity of the biological and cellular systems. In addition to this, the design of cavities and preparations to receive the restorations require a wear on the remaining healthy dental tissue [5]. If the wear is insufficient, the restoration can be dislodged, not achieving the necessary cervical seal, and alterations in the occlusion could give rise to alterations in the temporomandibular joint, periodontal or neuralgia. On the other hand, if the wear is excessive, pulp damage and even necrosis could occur. In addition, these types of restorations provide an aesthetic appearance.

Based on this, ceramics seem to be a better restoration option. They are aesthetically more like dental tissues. Manufacturers offer various options in terms of colors and handling, which can be used in different patients and have greater biocompatibility. This has made aesthetics and biocompatibility, characteristics that are the most desirable in restoration materials. That is why, dental zirconium restoration is considered an excellent restorative material. However, based on the hypothesis established in this paper and the results obtained, it is established that, although it is a good option, there are still other parameters that should be considered. Therefore, it is possible to conclude the following:

1. The mechanical behavior of dental zirconium, in fact, differs from that of dental tissues, its rigidity being the main factor to consider. The enamel and dentin, being one more elastic than the other, allow to perform its masticatory function without causing any failure in any of the tissues, mainly the enamel, since the dentin is able to withstand the hardness and rigidity of the enamel, in this way dissipating the loads and stresses generated outside the pulp tissue. With the restoration of the dental zirconium, this function is altered and the ability of the dentin to withstand the rigidity is overcome
2. So, in addition to seeking the aesthetics and biocompatibility of restorative materials, it is necessary to find their *biomechanical compatibility*, understanding this concept as the property of a material to resemble the mechanical properties of the tissues to be restored or replaced, in such a way that they imitate the functions of the lost tissue and allow their performance as nature designed them (mimicry). In turn, they allow the performance of the normal function of the surrounding tissues; above all these, a synergistic or symbiotic function is carried out such as that of the relationship between the enamel and dentin
3. This does not mean that the dental zirconium should be eliminated as an option for restoration material. However, it is necessary to carry out additional studies and find under what cases it is well indicated or if it is necessary to use it together with other materials to improve its functioning, that is, finding a material that solves its hardness and rigidity as does dentin with enamel

## Figures and Tables

**Table 1:** Mechanical properties used in the analysis.

Dental tissue	*Young's* modulus	*Poisson's* ratio dimensionless	Density
Enamel	70 GPa	0.30	0.25 g/cm^3^
Dentin	18.3 GPa	0.30	0.31 g/cm^3^
Pulp	2 GPa	0.45	0.1 g/cm^3^
Dental zirconium restoration	250 GPa	0.32	5.68 g/cm^3^

**Table 2:** Some details of the models.

	Healthy molar	Restored molar
Mesh	Tetrahedral solid elements	Tetrahedral solid elements
Meshing	Semicontrolled	Semicontrolled
Mesh quality	High-order quadratic elements	High-order quadratic elements
Nodes	129,005	363,380
Elements	74,907	246,254

**Table 3:** Comparison of the results obtained between both study cases in enamel.

Values	Case 1 (healthy molar)		Case 2 (restored molar)	
	Maximum	Minimum	Maximum	Minimum
Normal stresses in *X*	0.0025 Pa	-0.002525 Pa	-17.41 × 10^6^ Pa	8.56 × 10^6^ Pa
Normal stresses in *Y*	-0.0025 Pa	0.002453 Pa	-26.63 × 10^6^ Pa	5.60 × 10^6^ Pa
Normal stresses in *Z*	0.0096 Pa	-0.003641 Pa	-47.90 × 10^6^ Pa	6.07 × 10^6^ Pa
*von Mises* stresses	0.0124 Pa	0.0003 Pa	40.28 × 10^6^ Pa	0.08 × 10^6^ Pa

**Table 4:** Comparison of the results obtained between both study cases in the dentin.

Values	Case 1 (healthy molar)		Case 2 (restored molar)	
	Maximum	Minimum	Maximum	Minimum
Normal stresses in *X*	-0.0015 Pa	0.0013 Pa	-8.16 × 10^6^ Pa	3.69 × 10^6^ Pa
Normal stresses in *Y*	0.0015 Pa	-0.0012 Pa	-6.33 × 10^6^ Pa	4.19 × 10^6^ Pa
Normal stresses in *Z*	-0.0026 Pa	0.0025 Pa	-28.90 × 10^6^ Pa	3.00 × 10^6^ Pa
*von Mises* stresses	0.0027 Pa	0.00002 Pa	27.65 × 10^6^ Pa	0.53 × 10^6^ Pa

**Table 5:** Comparison of the results obtained between both study cases in the pulp.

Values	Case 1 (healthy molar)		Case 2 (restored molar)	
	Maximum	Minimum	Maximum	Minimum
Normal stresses in *X*	-0.0006 Pa	0.0004 Pa	4.69 × 10^6^ pa	-2.34 × 10^6^ Pa
Normal stresses in *Y*	-0.0007 Pa	-0.0004 Pa	4.79 × 10^6^ Pa	-2.43 × 10^6^ Pa
Normal stresses in *Z*	-0.0004 Pa	0.000402 Pa	11.01 × 10^6^ Pa	-5.91 × 10^6^ Pa
*von Mises* stresses	0.0007 Pa	0.000002 Pa	5.29 × 10^6^ Pa	3.003 × 10^6^ Pa

## Data Availability

The data used to support the findings of this study are included within the article.
